# Nitrous Oxide and Oxygen in Dental Surgery

**Published:** 1902-02-15

**Authors:** D. H. Ziegler

**Affiliations:** Cleveland, Ohio


### NITROUS OXIDE AND OXYGEN IN DENTAL SURGERY.

BY DR. D. H. ZIEGLER, D.D.S., CLEVELAND, OHIO.

Read at the Thirty-sixth Annual Meeting of the Ohio State Dental Society, December 3, 1901.

The year 1844 is memorable as having witnessed the greatest evolution in modern surgery. In this year Dr. Horace Wells demonstrated to the world that it was possible to perform minor surgery without the slightest pain being experienced. From that day up to the present time special attention has been given to improving the methods of admintstering nitrous oxide for the extraction of teeth.

My attention was first directed to this subject last June, when several of the leading surgeons of Cleveland were at their wits end to anesthetize a patient for an operation. The patient was in such a condition that chloroform and ether were out of the question. At this late day it was again necessary to call upon the dental profession to help them out of their dilemma. Dr. J. F. Stephan and his nitrous oxide and oxygen machine were pressed into service. For forty-five minutes I witnessed a perfect tyye of anesthesia. The success of the operation has left a profound impression on my mind.

The only excuse I have in presenting this paper is that it may bring it more universally before the notice of the members of this Society. The statements which I will present are not so much from my own observation as they are of men who have made a detailed study of this subject. As early as 1868 Dr. Andrews, of Chicago, published several cases in which by mixing nitrous oxide with oxygen he obtained a more satisfactory form of anesthesia; but, strange to say, his observations failed to attract the attention of the profession, and it was not until ten years later that Paul Bert again drew attention to the subject. His conclusions were that it was impossible to produce anesthesia with such a mixture without increasing atmospheric pressure, but it soon became clear from the observations of other investigators that such an increase was not absolutely necessary. The first successful attempt to administer it at ordinary atmospheric pressure was made by Dr. Hillischer, of Vienna, but I feel that much credit is due to Dr. Hewitt, of the Dental Hospital of London, for devising an apparatus which made it possible to administer the combination successfully, also for the scientific research which he has made in connection with the two gasses.

When nitrous oxide was first introduced, the necessity of excluding all atmospheric air was considered necessary to produce anesthesia; but from time to time cases were recorded in which by alternate inhalation of nitrous oxide and air it was found possible to maintain more or less complete anesthesia, until to-day it is never given pure by men who are considered authority on the subject. With the pure gas asphyxial phenomena rapidly follow. This occurrence is incidental and not necessary to produce true anesthesia, as will be pointed out. The asphyxial symptoms of an ordinary nitrous oxide inhalation become attenuated, or even altogether vanish, when oxygen is added in suitable proportions, and there can be no doubt that in employing the pure agent we are in a sense committing a physiological error. Why should the patient be subjected to an intercurrent state of asphyxia, when that state is not essential to the anesthesia? I shall endeavor to point out that with a proper percentage of oxygen we are able to get a more tranquil stage of anesthesia and obviate nearly all the unpleasant symptoms of pure nitrous oxide anesthesia.

Anoxæmic convulsions are prevented even with a small percentage of oxygen. During the inhalations of nitrous oxide with oxygen to 4 percent, some degree of anoxamic convulsions is very common; but when once 5 percent of oxygen is reached very little convulsive movement is observed, and with 6 percent and over there is no such movement visible.

With regard to alterations in the patient's color, we find that with less than 11 percent of oxygen some degree of lividity is present. With 8, 9 and 10 percent of oxygen the alteration is very slight. The effect of oxygen in preventing stertor is very marked; with percentages as small as 3, 4 and 5 the ordinary stertor of pure nitrous oxide loses its irregular character and becomes replaced by a regular snoring sound similar in its type to that of ether and chloroform, and as the percentage of oxygen is increased the snoring becomes less pronounced. With 20 percent of oxygen the snoring altogether vanishes. Phonated sounds are far less common under nitrous oxide and oxygen than under nitrous oxide and air; still, they are liable to arise if the percentages are too large or too small. With the proper proportion of oxygen reflex and excitement movements are uncommon, but are likely to assert themselves with too great a percentage of oxygen. As regards the general result, Hewitt gives the following tabulation: The mixture for adult males are those containing 5, 6 or 7 percent of oxygen, and mixtures containing 7, 8 or 9 percent are best for females and children.

*The administration:* In order to obtain good results careful attention must be paid to detail. It requires more skill to produce a good stage of anesthesia than with the pure gas, but if properly administered there is no comparison. In the first place the type of patient must be considered. The best subjects are middle-aged and elderly women; weakly, middle-aged men; young women are also good subjects. These patients are very easily anesthetized by following the general routine of administration. Young men of strong, robust constitution require considerable attention; if we increase the percentage of oxygen as rapidly in these cases as in the former, they may give trouble by reason of muscular spasms, but with the proper percentage of oxygen these symptoms are attenuated. Alcoholic subjects under nitrous oxide display a great tendency toward jactitation, but with a mixture rich in oxygen, which will make it necessary to prolong the administration, good results are to be obtained. Patients with adenoid growths, enlarged tonsils, easily develop asphyxial symptoms under pure nitrous oxide, but with considerable percentage of oxygen these patients can be anesthetized without submitting them to this physiological condition.

In the second place, make sure that you have a sufficient supply of the two gases on hand and that the apparatus is in perfect working order. The face piece must fit with perfect co-aptation so as to exclude all atmospheric air; an imperfect fitting face piece with pure nitrous oxide might not be detrimental, but in case of the mixture, it might lead to partial or complete failure. After adjusting the face piece and the administrator sees and hears that breathing is free, the indicator is turned to "2". Nitrous oxide with a small percentage of air, will thus gain admission to the lungs; after two or three seconds the oxygen indicator may be turned to "3", and in a few seconds more to "4." In about fifty seconds from the commencement of the administration, the indicator may as a general rule be moved as far as "5," and in twenty to thirty seconds more it may usually be allowed to point to "6," "7" or "8." In dental operations this is usually all the oxygen required to produce a tranquil stage of anesthesia. This mode of procedure is for a typical case; but as different subjects require a greater or less percentage of oxygen, the successful anesthetist is one who can pilot his patient along a narrow channel; he must be able to see at a glance what the future effect of an increase or decrease of oxygen will be.

For instance in children, anæmic subjects, and debilitated persons, we may increase the oxygen indicator more quickly; while in strong, robust, muscular men, the indicator must be moved much slower. On the other hand, should phonation, laughter, excited movement or struggling assert itself, the percentage of oxygen must not be increased. It may even be necessary to turn back the indicator for a few breaths, but not necessarily till all symptoms of excitement have subsided, knowing that they will subside in obedience to the lessened proportion of oxygen. The administrator must avoid the clonic respiratory movement, so common under pure nitrous oxide, which prevents a free and lengthy intake of the anesthetic; and again, any inconvenient signs of complete anesthesia.

The two bags must be kept as nearly as possible equal in size. It is rarely necessary to replenish the oxygen bag for a dental operation, but the nitrous oxide valve must be kept constantly working.

Generally speaking, surgical operations may usually be commenced in two and a half to three minutes after the face piece has been applied. And at this moment it is important that the patient's symptons should indicate a diminution rather than an excess of oxygen in the system.

When the typical nitrous oxide and oxygen anesthesia has become established, the patient's condition will be suggestive of a natural sleep, the respiration is remarkably calm and perfectly regular. The loud stertor of an ordinary nitrous oxide administration is never met with; the thoracic and abdominal movement so often witnessed under pure nitrous oxide are strikingly absent, owing to there being less venous engorgement, and hence less swelling of all parts within the upper air tract. The color of the face and lips varies; some patients become a trifle paler than usual, while in others the color becomes more florid. The duskiness and lividity so common under pure nitrous oxide, are, generally speaking, entirely absent. The pulse is invariably accelerated; but is neither so quick nor so small as the pulse under pure nitrous oxide.

Clonic muscular movements are very conspicuous by their absence, but a minor degree of tonic spasm in the muscles of the neck, back and extremeties may be manifested.

The pupils as a rule are of moderate size; wide dilation such as met with under nitrous oxide is very rare. The conjunctival reflex is, in the vast majority of cases. quite absent.

Anesthesia is known to be present by one or all of the following signs: (1) The conjunctival reflex is lost. (2) The breathing is regular and tranquil, or is softly snoring in character. (3) The arms are flaccid. (4) The eyeballs are fixed or present slight oscillatory movements.

The after effect is usually very satisfactory, but not quite so speedy as from nitrous oxide alone. The patient will be a trifle more dazed; nausea or actual vomiting, although rare, is more common than after nitrous oxide alone.

Dr. Hewitt in sixty-nine carefully timed operations, finds a gain of fourteen seconds in the average period of resulting anesthesia. By comparing the pure nitrous oxide anesthesia with a mixture of oxygen everything points in favor of the latter.

We are able to obviate nearly all the unpleasant effects of the pure gas; our patient at all times presents a more natural appearance, and an average gain of fourteen seconds is certainly worth considering.

The question will naturally arise, can not the same results be accomplished with atmospheric air? We have men with us who no doubt will say they can, and I honestly believe that they are sincere when they make those statements; but their statements may be merely from a local observation, and not from a purely scientific investigation. Before we criticise or condemn a method, I feel that we ought to do so only after studying both sides of it. Cases are recorded in which general surgical operations lasting an hour or more have been performed under nitrous oxide and air, but I will try to show that there is little to be said in favor of this mixture, as compared with oxygen. So let us draw our comparison from Dr. Hewitt's scientific investigation of 106 cases.

He employed a specially made gasometor by which he could administer any desired percentages of nitrous oxide and air. He found that anesthesia could be obtained provided the latter did not exceed thirty percent. With thirty-three and one third percent of air he failed to produce complete anesthesia. With small percentages of air the symptoms were practically identical with those produced by the pure gas. The shortest anesthesia was met with when employing three percent of air (30.3 sec's.) and thirty percent of air (29.7 sec's.). The longest average anesthesia being recorded with 14, 16 and 22 percent of air. It is an interesting fact that there was more anoxæmic convulsions with three and five percent of air than with pure nitrous oxide. While the nitrous oxide and oxygen experiment showed that with five percent of oxygen very little convulsive movement was observed. Lividity and cyanosis were also more evident with small percentages of air than with pure nitrous oxide, and there was no marked change until thirty percent of air was reached. With this percentage he had an average operating period of 29.7 sec. Compare this with six percent of oxygen, we have a very slight lividity and an average operating period of forty-four seconds, or a gain of 14.3 seconds.

There was less phonation under nitrous oxide with small percentages of air than under nitrous oxide itself; but with a mixture containing more than ten percent of air phonation was common. While under nitrous oxide and oxygen phonated sounds are far less common than under nitrous oxide and air. Out of 153 cases he noted it more or less in twenty-nine. So far as the general result of his investigation, it showed that with percentages of air between fourteen and twenty-two a very decided improvement was manifested over the pure gas, but this percentage is not large enough to eradicate lividity and cyanosis, nor phonated sounds. Reflex and excitement movements are less marked but not entirely done away with. All these symptoms referred to can be prevented with the proper percentage of oxygen, and still produce tranquil anesthesia.

The experiment showed that with eight, nine and ten percent of oxygen and alteration of the natural features were very slight, or, in other words, there was no lividity, and if we mix with nitrous oxide a percentage of air which will prevent this condition and other symptoms referred to, we shall in most cases also prevent deep anesthesia. For example, to obtain eight percent of oxgen, we will require a mixture of forty percent of air; this would give us a combination of sixty percent of nitrous oxide, eight percent of oxygen and thirty-two percent of nitrogen. The eight percent of oxygen would be sufficient to preserve the natural color of the patient's face and prevent muscular spasm, but the sixty percent of nitrous oxide would be insufficient to produce tranquil anesthesia. By employing oxygen for oxygenating purposes, we are able to replace the thirty-two percent of useless nitrogen, by a quantity of useful nitrous oxide, and the proportion will now be ninty-two percent of nitrous oxide. and eight percent of oxygen.

I feel safe in saying that there is no anesthetic at present known which is so devoid of danger as that which results from this mixture.

DISCUSSION.

Dr. Cassidy, of Covington, Ky.: In opening this discussion I must say that I agree with the essayist in all he has said. I find no fault with any point made in his paper. It has been more than twenty years since I practiced giving nitrous oxide gas for anesthesia, but every year during that interval I have investigated the chemical nature of that gas, and am more convinced of and confirmed in the truth of the statement that nitrous oxide has no inherent power within itself, and that its remarkable energy is due to its oxidizing power. It is generally conceded by all authorities that nitrous oxide, when inhaled, passes through the arterial circulation unchanged. In that way it supersedes the normal function of the iron in the blood; but when it reaches the capillaries, where the processes of metabolism occur, where there are oxidizable substances, decomposition occurs just as sure as two and two make four; otherwise our knowledge founded on experiment, no theory about it, is entirely wrong. A recent text-book says, without any qualification at all, or any proof of the statement, that there is no separation of the elements of nitrous oxide at the temperature of the human body. Such a statement is remarkable to me. If any of you have made nitrous oxide and collected it over old rain-water, as I have done many a time—in fact I have used putrid water, so putrid that you could smell it several steps away—you will agree with the fact that such water, after absorbing nitrous oxide, in a short time becomes perfectly sweet. No doubt others of you have noticed the same fact. This change is accomplished by the oxygen set free by the decomposition of nitrous oxide in that water, even at a temperature of 50° F. There is no question in my mind but that the gas decomposes. At the Tri-State Meeting the opinion was expressed by one speaker that nitrous oxide acted as an anesthetic as such, and did not decompose in the body; and this, too, when it is known that all products of chemical action with nitrous oxide are oxidation products. So I think I am warranted in making the above assertion.

In reference to the action of oxygen or of air, both act chemically in the same way, but free oxygen is more powerful than the same volume of air. Oxygen is not handicapped by the mixture of other gasses, while air is. One volume of oxygen is equal to five volumes of air. The chemical action of the oxygen in air is, however, the same as that of free oxygen.

Now you say, if nitrous oxide gives up oxygen, why mix oxygen with it to prevent the cyanosis? Nitrous oxide supersedes iron in the arterial circulation, and you give oxygen with it, which stimulates natural respiration by uniting with the iron, which is carried to the capillaries, where oxygen is given up and carbon dioxide is formed rapidly and abundantly. This iron serves the purpose of then carrying out the excess of carbon dioxide. This is the science of it, and I am sorry the paper did not allude to this phase of the subject. Dr. Watt has been quoted to-day in regard to hæmophilia. Gentlemen, you are always safe in quoting what he said when he was in his prime. I heard him say, in 1868, that he gave air with nitrous oxide in nearly all cases, in fact almost made a specialty of it. I have heard him describe how he took nitrous oxide for an operation on his own person, and used air in sufficient quantity to prevent cyanosis, and that he was perfectly aware of what was going on, but suffered no pain, When people talk of perfect anesthesia it does not necessarily mean unconsciousness.

Dr. Stephan, of Cleveland, Ohio: I am very much interested in the combination of these two gases as an anesthetic. I have been using it in my practice for the past year and have had the pleasure of using it in several extended surgical operations. In the case mentioned in the paper the patient's heart lost one-half the normal beat and had anesthesia for forty minutes, with a stronger pulse and good respiration. The surgeons were surprised at the results and we were gratified.

They were surprised that there was no surgical shock after the operation; the heart maintained its strength throughout the operation and has been strong ever since. There is less danger from shock under these two gases than there is under any other anesthetic used to-day. This is a point which it is well to make a note of. The anesthesia under nitrous oxide and oxygen is as much greater than the anesthesia under nitrous oxide and air, as nitrous oxide and air is greater than that of pure nitrous oxide. In the administration of nitrous oxide and oxygen you should begin with a low percentage of oxygen, because of the oxygen already normally in the lungs; and, by gradually increasing it so as to maintain the normal amount of oxygen in the blood, we are then able to continue the anesthesia almost indefinitely. The longer the anesthetic condition is continued, the greater the amount of oxygen the patient is able to take, and should take. I believe this anesthetic will be used by surgeons to the great exclusion of chloroform and ether in the future.

Dr. Wilson, of Cleveland, Ohio: I have seen nitrous oxide and oxygen administered in a few cases, and feel that it is a coming thing with us. I trust it will be, so that Dr. Ziegler will be able to make an exhibit before we leave this meeting. I would suggest that the gas be administered immediately after a meal, as it is more liable to cause sickness and irritation on an empty stomach. Patients are not so liable to sickness with nitrous oxide as with nitrous oxide and oxygen, because nitrous oxide acts very quickly and the patient is not made sick; with nitrous oxide and oxygen the anesthetic stage does not come on for some time, and the patient has taken at least three times as much nitrous oxide, so there is more danger of sickness. By the use of nitrous oxide I have never had a sick patient in my practice, but with nitrous oxide and oxygen I have seen several. It is very gratifying, however, to notice patients while under the anesthesia of nitrous oxide and oxygen. They are perfectly quiet; no such spasms as seen with nitrous oxide, and the color is most excellent. In fact, the patient looks as if he were sleeping.

Dr. Ambler, of Cleveland, Ohio: I have had no personal experience with this mixture, but have seen it administered several times. Its effects were truly wonderful and as good as any person could expect or ask for. In all the administrations I have seen there were no bad results or symptoms. Dr. Wilson said he never saw a patient sick from the use of nitrous oxide, but I have. Have not administered nitrous oxide very largely, but can remember very well several bad results.

Dr. Zeigler in closing the discussion; There is very little more to say in connection with this combination. Really after working with it, the results are so satisfactory, that the more one sees of it the more enthusiastic he becomes. The operation I made reference to in the paper was, indeed, quite wonderful, at no time did the heart drop below sixty and the pulse rate was increased or decreased at will by increasing or decreasing the amount of oxygen thus showing its effect upon the weakened heart. One disadvantage probably in the use of this mixture is that it is necessary to have an assistant to help administer it. The S. S. White Dental Manufacturing Co. has an apparatus on exhibition here in the other room. If you examine it you will readily see the reasons for an assistant. It requires one person to take charge of the apparatus. It is necessary to keep the tension of both gas bags at about the same pressure at all times. It is very important to keep perfect control of the oxygen valve as the introduction of too much oxygen into the system will bring on a state of excitement, which may be followed by muscular spasms. The operator of course must devote his whole attention to the patient and his assistant must control the supply of gas. You have all seen those cases of muscular spasms from the administration of nitrous oxide where it required two or three men to hold the patient in the chair. I have never seen any such cases under gas and oxygen, the patient is at all times under perfect control.

